# Biochars from Lignin-rich Residue of Furfural Manufacturing Process for Heavy Metal Ions Remediation

**DOI:** 10.3390/ma13051037

**Published:** 2020-02-25

**Authors:** Baobin Wang, Miao Ran, Guigan Fang, Ting Wu, Yonghao Ni

**Affiliations:** 1 College of Light Industry Science and Engineering, Nanjing Forestry University, Nanjing 210037, China; 2 Key Lab. of Biomass Energy and Material, Institute of Chemical Industry of Forestry Products, CAF, Nanjing 210042, China; 3 Limerick Pulp and Paper Centre and Department of Chemical Engineering, University of New Brunswick, Fredericton, NB E3B 5A3, Canada

**Keywords:** lignocellulosic residue (LCR), biochar (BC), adsorption

## Abstract

The pentose/furfural industrial manufacturing process uses corn cob residue as a raw material, where such a process yields significant amount of lignin-rich residue (LCR) at the end, which is commonly disposed by burning. In this study, the conversion of LCR to biochars (BCs), and their subsequent applications for heavy metal ion removal, were investigated. The BCs were prepared through hydrothermal carbonization and post-activation, using either ZnCl_2_ or H_3_PO_4_ treatment. The as-prepared activated BCs were characterized using N_2_ adsorption–desorption isotherms, XRD, FT-IR, SEM and TEM, and their performance in removing heavy metal ions (Pb^2+^, Cu^2+^, Cd^2+^) from aqueous solutions was assessed. The ZnCl_2_-activated BCs (BC-ZnCl_2_) exhibit a higher adsorption capacity than the H_3_PO_4_-activated BCs (BC-H_3_PO_4_), mainly due to the differences in their chemical/physical characteristics. The related adsorption kinetics and isotherms were analyzed.

## 1. Introduction

In China, there are many commercial operations for extracting pentoses/furfural from corn cobs [1,2,3], and in the process, solid lignocellulosic residue (LCR), are generated at approximately 23 million tons annually [4]. Currently, the generated LCRs are burned, which is an undesirable solution; on the other hand, by following the integrated biorefinery concept [4,5], other value-added products from LCR may be produced. Accordingly, there have been many studies reported on the topic of converting LCR to useful products, including the production of porous carbon for super capacitor electrodes [6], hydrochars [7], phenols [8] and biofuels [9,10]. These studies show promising LCR-derived products that can bring alternative values to current burning practices.

Carbonaceous adsorbents (CAs) exhibit various potential applications, which include adsorption [11], catalysis [12,13], energy storage [14] and biomedicine [15]. Among them, water purification is one application that has received much attention, and is of particular interest in China [16]. CAs have favorable adsorption characteristics, e.g., high surface area, specific pore volume and surface functional groups [17]. However, the specific properties of CAs are largely dependent upon the particular activation process applied in their preparation [18,19]. Most often, ZnCl_2_ and H_3_PO_4_ are used for the chemical activation process, which leads to the formation of chars with high porosity and surface area, thus imparting high adsorption capacity [20,21].

Many studies have been conducted regarding the removal of toxic heavy metals from water using various techniques, such as adsorption, ion exchange, membrane filtration, electrodialysis, reverse osmosis, ultrafiltration [22] and photocatalytic degradation [23]. Among them, adsorption, specifically using carbonaceous materials, is effective for water purification. In addition, by utilizing biomass residues, added value is brought to the overall process, which in turn minimizes costs, and provides an alternative method for treating solid waste [24].

In this study, a new process was developed aimed at the value-added conversion of LCRs to BCs. The process consisted of two stages: (1) a hydrothermal stage to produce hydrochar, and (2) an activation stage to obtain low-cost biochars. The hydrothermal stage was conducted under inert atmospheric conditions at 250 °C for 2 h. The activation process was performed using activating agents (H_3_PO_4_, ZnCl_2_) at 500 °C for 2 h. Characterization of the as-prepared biochars was performed via N_2_ adsorption and desorption analysis, Fourier-transform infrared spectroscopy (FT-IR), the Boehm titration method, and X-ray diffraction (XRD). In addition, the as-prepared biochars were further tested for performance in removing Pb (II), Cd (II) and Cu (II) from aqueous solutions, and the adsorption parameters, i.e., solution pH, initial concentration, and contact time on adsorption capability, were investigated. Finally, the equilibrium isotherms and adsorption kinetics were developed.

## 2. Materials and Methods

Materials and Chemicals. Lead (II) nitrate (≥99.99%), copper (II) nitrate (≥99.99%) and cadmium (II) nitrate (≥99.99%) were purchased from Macklin Inc. (Shanghai, China). All materials and chemicals were used as received, without any further treatment, and ultrapure water was obtained from a Hi-tech (Beijing, China) water filtration station (18.2 MΩ·cm at 20 °C). The pHs of working solutions were adjusted with 0.1 M NaOH and 0.1 M HNO_3_.

Material Synthesis. LCR generated from the furfural production process was provided by Shandong Longlive Biotechnology Company (Dezhou, China), and was then used to prepare biochars (the BC-H_3_PO_4_:H_3_PO_4_ biochar and the BC-ZnCl_2_:ZnCl_2_ biochar). The chemical composition (mass basis) of the CCR consisted of: cellulose (72.3%), lignin (16.2%), hemicellulose (7.2%), extractives (8.1%) and ash (4.2%), and was determined in accordance with TAPPI standard methods (T 201, T 222, T 223, T 204 and T 211, respectively).

**Figure 1:**
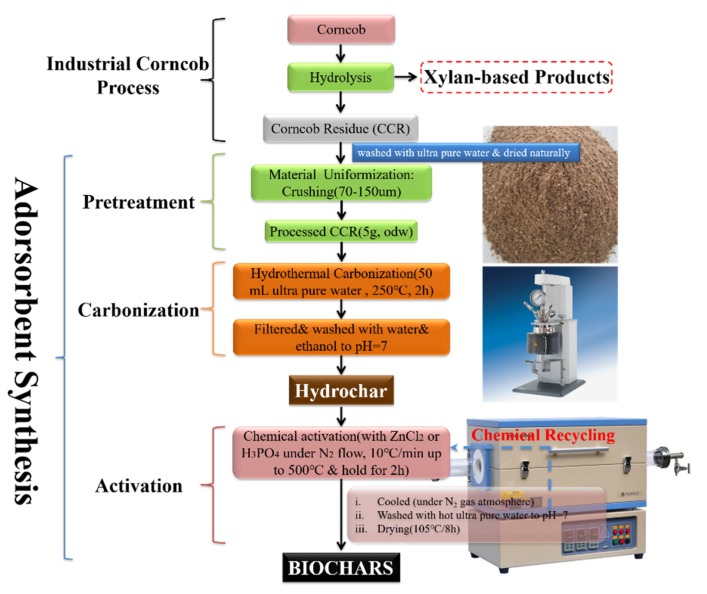
Synthesis procedures for preparing biochar (BC) samples: BC-ZnCl_2_ and BC-H_3_PO_4_.

As depicted in Figure 1, the preparation of the biochar material was performed using three steps, these being pretreatment, carbonization and activation.

Pretreatment. Initially, the LCR was dried to a constant weight under room temperature, and subsequently milled to a particle size of below 100 mesh (0.15 mm), sieved, and then stored in a sealed bag.

Carbonization. The hydrothermal carbonization process was performed according to the following procedures [7]: 5 g (odw) of LCR and 50 mL of distilled water were loaded into a 250 mL Parr stirred pressured reactor (4576A, Parr instrument company, Moline, IL, USA). Next, the reactor was sealed, flushed with N_2_, and heated to 250 °C for 2 h to carbonize the sample. These carbonized samples were then removed from the reactor and washed with ethanol and water until the wash filtrate was neutral. The final product obtained from the carbonization process was denoted as “hydrochar”.

Activation. The hydrochar was mixed with an aqueous solution of 40% H_3_PO_4_ with the ratio of hydrochar/H_3_PO_4_ at 1:6 (kg/kg), allowing the treatment for 24 h at room temperature. Subsequently, the mixture was transferred to a tube furnace, which was flushed using N_2_. The mixture was then heated at a rate of 10 °C/min, until the desired activation temperature reached 500 °C, and this was maintained for 2 h, followed by cooling inside the furnace to room temperature. After cooling, the activated mixture was washed several times with hot, distilled water (60 °C) until a neutral pH was achieved. After washing, the sample was then dried at 105 °C in an oven for 8 h, and was labeled as BC-H_3_PO_4_. 

To prepare the product BC-ZnCl_2_, hydrochar was activated instead using ZnCl_2_ in a similar fashion as to the BC-H_3_PO_4_ product. To do this, the hydrochar was mixed with an aqueous solution of 40% ZnCl_2_ with a ratio of 1:10 (kg/kg), and placed into a cupel for a duration of 24 h at room temperature. The subsequent heating of the sample was performed identically to the heating process performed for BC-H_3_PO_4_. The activated product was then boiled with a volume of 1 M HCl equaling 10 times the volume of the hydrochar for a duration of 1 h. Next, the activated mixture was washed several times with hot, distilled water (60 °C) until the filtrate was absent of chloride ions. The sample was then dried at 105 °C in an oven for 8 h after washing, and was labeled BC-ZnCl_2_.

### 2.1. Adsorption Experiment

The effect of the solution’s pH was investigated by adjusting the starting pH of the metal ion solution prior to commencing the experiment. Initially, a metal ion concentration of 1.0 mM was prepared and allowed to reach equilibrium over a duration of 10 h. The initial pH of the solution was then adjusted within a pH range of 2.0 to 6.0 by adding appropriate amounts of 0.1 M NaOH or 0.1 M HNO_3_. The maximum tested pH value was fixed at 6.0 to avoid the precipitation of metal ions that can occur at higher pH values.

Equilibrium experiments were conducted at isothermal conditions of 25 ± 2 °C as a batch process. To develop adsorption equilibrium isotherms, 0.6 g of BC samples were placed into 250 mL Erlenmeyer flasks containing 100 mL of metal ion solutions, having different initial concentrations within the range of 0.5 to 5.0 mM for Pb (II), Cd (II) and Cu (II) metal ions at optimal pH conditions. The flasks containing the BC samples and metal ions were then continuously agitated at 6.67 Hz for 10 h. The samples were then filtered through a 0.45 mm cellulose filter paper, and metal concentrations of the filtrate were analyzed. Metal ion concentrations within solutions were analyzed using the atomic absorption spectrum (Shimadzu AA6701F, Kyoto, Japan).

### 2.2. Characterization of Carbon Materials

The crystallinity of samples was determined using X-ray diffraction (XRD) (D8-ADVANCE, Bruker, Bremen, Germany) with Cu Kα radiation at 10 kV. The XRD data was collected from 10 to 80° (θ) at a scan rate of 2°/min. The surface area was calculated using the Brunauer–Emmett–Teller (BET) method. The pore volume was determined from the amount of N_2_ adsorbed at P/P_0_ = 0.98. The surface functional groups and adsorptive form of heavy metals were identified using IR spectroscopy. The reflectance infrared spectra were obtained using a Shimadzu FT-IR spectrophotometer (Kyoto, Japan), where each spectrum was recorded over a wavenumber range of 500−4000 cm^−1^. Oven-dried KBr pellets were used for sample preparation.

## 3. Results

### 3.1. Concept of Preparing Biochars from LCR for the Removal of Heavy Metal Ions

**Figure 2:**
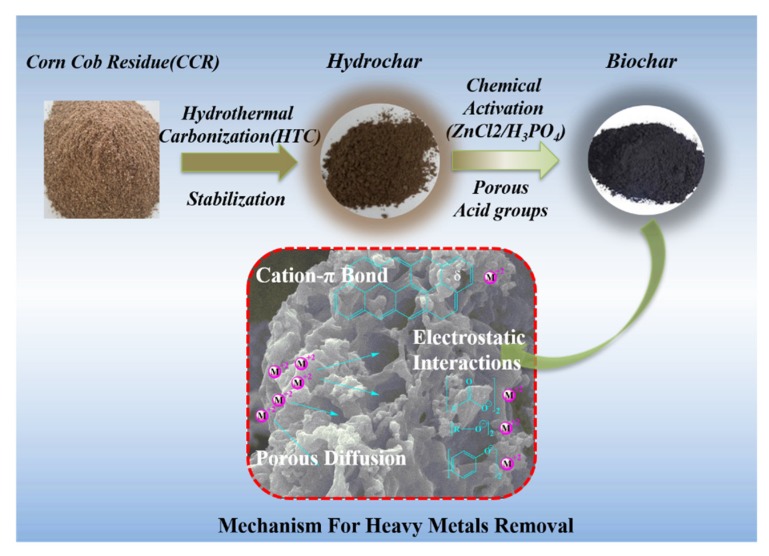
Concept of Biochars preparation and their applications for heavy metal ion removal through adsorption mechanisms.

As illustrated in Figure 2, LCR was hydrothermally carbonized (250 °C, 2 h) first to hydrochar. Subsequently, the hydrochar was chemically activated using either H_3_PO_4_ or ZnCl_2_ as the activating agent. Finally, the biochar products were used for metal ion removal from aqueous solutions through an adsorption mechanism. 

A high surface area and porous structure of the biochar are key parameters for efficient metal ion removal based on the adsorption concept. A porous structure allows metal ions to easily diffuse within the biochar, which facilitates the adsorption process. Moreover, as metal ions diffuse into the porous network, a high surface area improves the availability of functional groups for metal ions interaction. There are two main mechanisms of adsorption that occur in this process: (a) the acid groups (carboxyl (–COOH), phenolic groups (R–OH)) of the biochar will interact with the metal ions; and (b) the aromatic structures formed in the biochar due to graphitization can act as π donors, and as a result, cationic metal ions will interact with the aromatic rings (i.e., through the cation–π interactions). Both mechanisms play an important role in the metal ion removal during the sorption process. 

### 3.2. Physicochemical Characterizations

**Figure 3:**
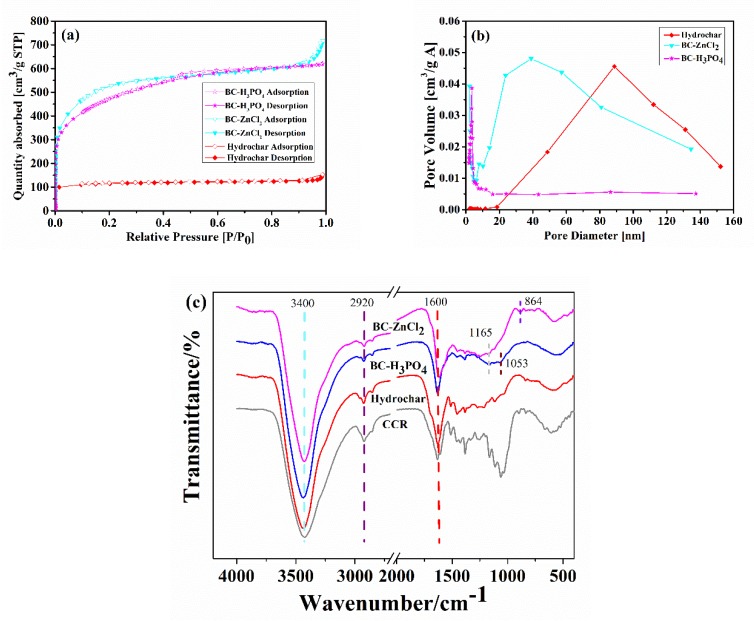
Physicochemical properties of the Biochars. (**a**) Nitrogen adsorption and desorption isotherms of Hydrochar, BC-H_3_PO_4_ and BC-ZnCl_2_ at 98 K; (**b**) Pore size distribution of Hydrochar, BC-H_3_PO_4_ and BC-ZnCl_2_; (**c**) FT-IR spectra of CCR, Hydrochar, BC-H_3_PO_4_ and BC-ZnCl_2_.

The nitrogen sorption isotherms of hydrochar and the prepared bio-based activated carbons are presented in Figure 3a. The N_2_ adsorption/desorption isotherms of the BC-H_3_PO_4_ and BC-ZnCl_2_ samples resemble the typical Type I isotherm, and a plateau occurs at low relative pressure with a predominance of micropores in the sorbent, followed by continuing N_2_ adsorption into the mesopores once the micropores are saturated [25,26]. The N_2_ isotherms also demonstrate a small hysteresis loop in the desorption curve, which implies the presence of mesopores, with the possible occurrence of a capillary condensation phenomenon.

The pore distribution of the hydrochar, BC-H_3_PO_4_ and BC-ZnCl_2_ samples are shown in Figure 3b. The pore size distribution curve of BC-H_3_PO_4_ shows a microporous structure, while the combined micropores/mesopores are for the BC-ZnCl_2_ sample. 

The BET surface area and pore volume of the hydrochar, BC-H_3_PO_4_ and BC-ZnCl_2_ samples are shown in Table 1. The results show that the H_3_PO_4_ and ZnCl_2_ treatments during the activation process are effective for producing activated carbons with increased high surface area (790 m^2^/g for BC-ZnCl_2_ and 680 m^2^/g for BC-H_3_PO_4_) and high pore volume (0.74 cm^3^/g for BC-ZnCl_2_ and 0.65 cm^3^/g for BC-H_3_PO_4_). Interestingly, the BC-ZnCl_2_ sample demonstrated higher BET surface area and pore volume compared to the BC-H_3_PO_4_ sample. The results from the BET analysis correlate well with those found in the literature regarding H_3_PO_4_ and ZnCl_2_ activating treatment. For example, Myglovets et al. reported that the surface area of lignosulfonate-based carbons does attain 1370 m^2^/g after phosphoric acid activation at 1000 °C [27]. In addition, a tomato processing waste-based char was obtained with high BET surface area (1093 m^2^/g) after zinc chloride activation [28].

The functional groups on the surface of the biochars were determined using the Boehm titration method; the results are presented in Table 1. Clearly, the BC-ZnCl_2_ sample had more functional groups than the BC-H_3_PO_4_ sample. The acidic groups (carbonyl, carboxylic, lactones and phenol groups) content in the BC-ZnCl_2_ and BC-H_3_PO_4_ samples were 0.98 and 0.69 mmol/g, respectively. It was reported that 50% H_3_PO_4_ and 75% ZnCl_2_ impregnation generates approximately 5.6 mmol/g and 2 mmol/g acidic groups, respectively, in the activated carbon [29].

FT-IR characteristics of the raw material (LCR), hydrochar and the BC-ZnCl_2_ and BC-H_3_PO_4_ samples, are shown in Figure 3c. The CCR samples showed similar features to regular lignocellulosic materials: hydroxyl groups (∼3400 cm^−1^), C–H stretching from –CH_2_ groups (2920 cm^−1^), C–O stretching (1000–1350 cm^−1^), C–O–C stretching (∼1164 cm^−1^) and glycosidic linkages (∼895 cm^−1^) [30]. After the hydrocarbonization process, many of these functional groups disappeared. The biochar samples showed a wide band at approximately 3400 cm^−1^, which can be associated with the OH stretching vibration mode in alcohol and phenol.

For the BC-ZnCl_2_ sample, the band located at about 1600 cm^−1^ is attributable to C=C vibrations, those at 1720 cm^−1^, 1165 cm^−1^ and 864 cm^−1^ are due to C=O, C–O, and C–H vibrations, respectively [31]. For the BC-H_3_PO_4_-hydrochar sample, the bands at 1160–1180 cm^−1^, 1060 cm^−1^ and 950–990 cm^−1^ are attributed to the presence of phosphorus species [27]. The band at 1720 cm^−1^ is assigned to the stretching mode of carboxylic bonds [32]. Those at around 1150–1180 cm^−1^ can be assigned to C–O stretching vibrations in a C–O–P (aromatic) linkage, to stretching vibrations of hydrogen-bonded P=O groups of phosphates or polyphosphates, and to P=OOH. The band at 1053 cm^−1^ may represent the P–O–P vibrations. FT-IR results demonstrated that these activated biocarbon samples are rich in the functional groups. 

**Figure 4:**
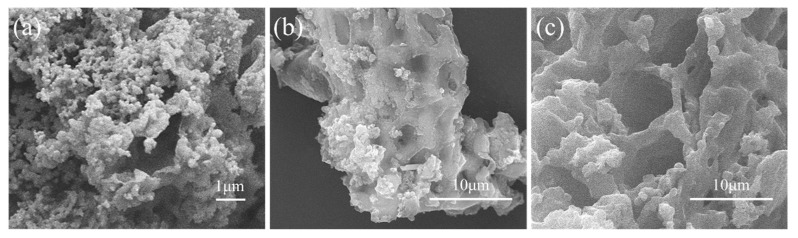
Scanning electron microscopy (SEM) images of (**a**) 500× Hydrochar; (**b**) 5000× BC-H_3_PO_4_; (**c**) 5000× BC-ZnCl_2_.

The surface morphology of hydrochar, BC-H_3_PO_4_ and BC-ZnCl_2_ samples are shown in the SEM images (Figure 4). The melted surface and irregular pores were evident for the hydrochar sample, while BC-ZnCl_2_ and BC-H_3_PO_4_ samples exhibited heterogeneous, well developed porous structures. Comparatively, Angin found well-developed, porous structures for the ZnCl_2_-activated biochars that were produced from sour cherry stones [33]. Lastly, in accordance with the XRD pattern (Figure S1), the peaks at 26° and 43° are assigned to disordered graphitic (002) and (10) plane, respectively. The as-prepared biochar samples demonstrated typical broad peaks, indicating that the samples have a low crystalline degree.

**Figure 5:**
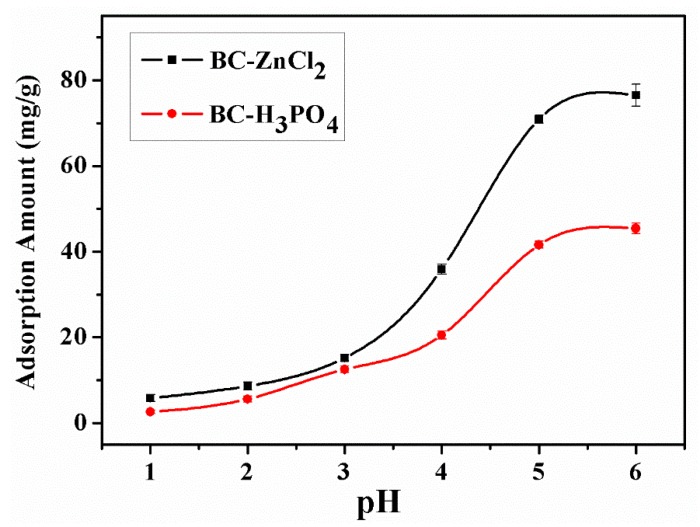
Adsorption amount of Pb^2+^ with various pHs.

The point of zero charge (pH_pzc_) (Table 1) was determined by analyzing the zeta potential charge when varying the pH range from 2 to 8. As shown, for the LCR sample, the pH_pzc_ is 9.8, while the pH_pzc_ of BC-H_3_PO_4_ and BC-ZnCl_2_ are 3.5 and 4.6, respectively. These results confirm the presence of these acid groups on the surface of activated carbons. Figure 5 exhibits the adsorption of Pb^2+^ under various pH. The adsorption amount of Pb^2+^ increased when the pH increased. The maximum adsorption amount (75.3 mg/g) was achieved at the pH of 5–6. When the pH is over 6, the metal precipitation would dominate, thus the adsorption of metal ions should be controlled [34]. The adsorption amount was minimal at low pHs (1–3), while the adsorption increased at elevated pHs (4–5). The metal ions will replace protons from the biochar at elevated pHs. When the pH is higher than the pH_pzc_, the surface of the sorbent is negatively charged, and attractive forces increase between the sorbent surface and metal ions. Furthermore, a higher pH favors the deprotonation of the sorbents’ phenolic and carboxylic acid groups, and thus, negative sites are created on both BC-ZnCl_2_ and BC-H_3_PO_4_ surfaces, thereby improving the adsorption of metal ions onto the surface of the sorbents. Mohan et al. [34] reported that the final pH of the solution is higher than the initial pH under acidic conditions, as neutralization and sorption are parallel processes; therefore, a pH of 5 was chosen as the optimal pH for performing the adsorption process. 

## 4. Discussion

### 4.1. Adsorption Kinetics

**Figure 6:**
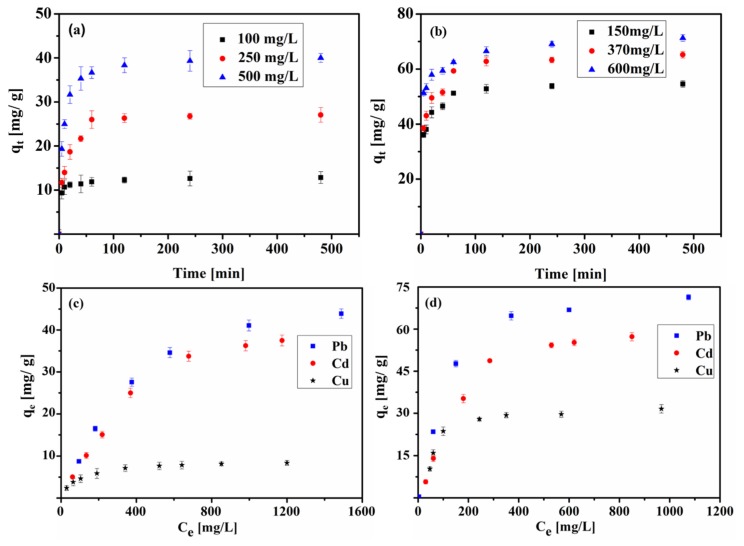
Kinetic analyses of Pb (II) adsorption on (**a**) BC-H_3_PO_4_ as a function of contact time; (**b**) BC-ZnCl_2_ as a function of contact time; Adsorption isotherms of Pd (II), Cd (II) and Cu (II) on (**c**) BC-H_3_PO_4_ and (**d**) BC-ZnCl_2_.

Figure 6a,b presented the adsorption analyses of Pb (II) onto the two BC samples. The process was very fast, and during the first 30 min, the uptake of Pb (II) was over 90% of the equilibrium amount, and the equilibrium was attained at approximately 4 h. Fast adsorption was also observed for the adsorption of Cd (II) and Cu (II) in the first 30 min (Figure S2). In the literature [35] it was reported that activated carbon prepared from marine green algae demonstrates high adsorption rates, and more than 90% of the equilibrium amount of Pb (II) ions are removed within the first 30 min. Furthermore, there are two adsorption kinetic regions during the adsorption process. Initially, there is a high adsorption rate region, which is associated with the large number of available active adsorption sites, and the high driving force facilitates the rapid adsorption of metal ions onto the active sites. The slow second region is due to the metal ion diffusion into the porous structure. 

The adsorption kinetics of heavy metal ions is related to the physical and/or chemical properties of activated carbons. Three models, namely, the pseudo-first-order (PFO), pseudo-second-order (PSO) and intra-particle diffusion model (IPD), were applied to the kinetic data. The fitting parameters associated with the three models for Pb (II) are presented in Table 2. 

Fitting parameters for Cd (II) and Cu (II) were also analyzed, as shown in Tables S1 and S2. The results show that the experimental data agrees with the PSO model (R^2^ near unity). In addition, the predicted values (Q) obtained from the PSO model are in accordance with the experimental *q*_exp_ values. 

### 4.2. Adsorption Isotherms

The adsorption isotherms of heavy metal ions are shown in Figure 4c,d (at 25 °C). For the BC-ZnCl_2_ sample, the adsorption uptake of Pb (II), Cd (II) and Cu (II) are 23.1–72.1 mg/g, 6.8–55.6 mg/g and 8.2–30.5 mg/g, respectively, which is highly dependent upon the initial concentration of these metal ions.

In comparison, Barczak et al. reported that heavy metal ion (Pb, Zn, Cu, Cd) adsorption isotherms for well-ordered carbons obtained from the hard templating of SBA-15 shows dependency on the initial concentration of the solution [36]. Additionally, the adsorption isotherms show an L-type according to Giles’s classification [37], indicating high adsorbate–adsorbent interactions.

The Freundlich (Equation (1)) and Langmuir (Equation (2)) isotherm models were used to fit the adsorption equilibrium experimental data.

(1) $$lnq_{e}=lnK_{F}+lnC_{e}/n$$

(2) $$C_{e}/q_{e}=1/q_{m}K+C_{e}/q_{m}$$

where *C_e_* is the metal ions concentration (mg/L) at equilibrium, *q_e_* is the adsorption capacity at equilibrium (mg/L), *K_F_* (L/mg) is the Freundlich constant, *1/n* is the heterogeneity factor, and *K* (L/mg) and *q_m_* (mg/g) are the Langmuir coefficients, representing the adsorption equilibrium constant and the monolayer capacity, respectively.

The Langmuir isotherm assumes monolayer sorption on a surface with a finite number of identical sites and uniform adsorption energies [38], while the Freundlich isotherm assumes that the adsorption occurs on a heterogeneous surface by multilayer adsorption, and the amount of adsorbate adsorbed increases infinitely with the increase of its concentration [39]. Table 3 depicts the fitting data of the equilibrium experimental data. The Langmuir isotherm gave a stronger fitting correlation constant (R^2^) in the range of 0.98–1.00, which is compared to that of the Freundlich isotherm in the range of 0.73–0.96. The BC-ZnCl_2_ sample exhibited a better adsorption capacity than the BC-H_3_PO_4_ sample for the three metal ions studied. These results are in agreement with those from the Boehm titration method, in that more acid groups facilitate electrostatic interactions, thereby increasing the adsorption capacity. 

The relative adsorption tendency can be quantified by the separation factor, *R_L_* as defined in Equation (3) [40].

(3) $$R_{L}=1/{(1\;+\;K}_{L}C_{0})$$

where *K_L_* is the Langmuir constant (L/mg) expressed in Equation (1), and *C_0_* is the initial metal ion concentration (mg/L). It is well known that values in the range of 0 < *R_L_* < 1 represent favorable adsorption [41]. The fitted results at equilibrium yield the *R_L_* values in a range of 0.002 to 0.495, indicating favorable adsorption performances for the as-prepared adsorbents.

**Figure 7:**
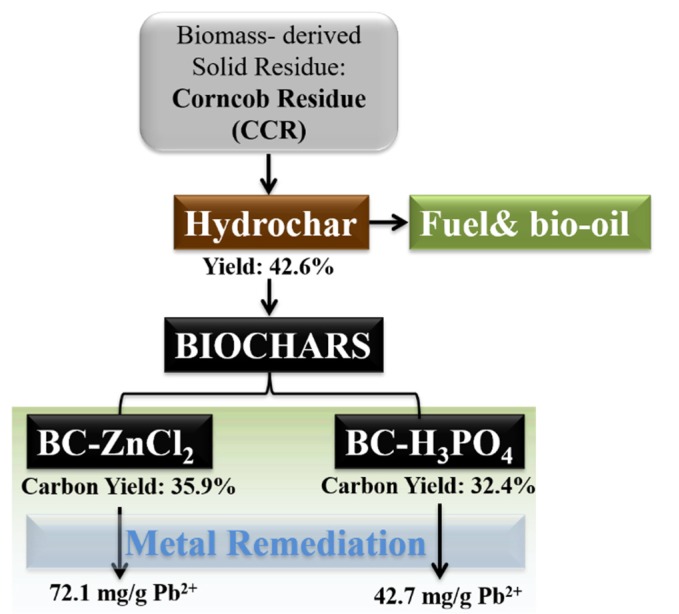
Concept of the biomass-derived biochar for Pb^2+^ remediation.

The concept of the biomass-derived biochar preparation for heavy metal ions removal is shown in Figure 7. In our previous study, corn cob residue was first utilized for the preparation of hydrochar through hydrothermal carbonization, which can be utilized for fuel, and then bio-oil can be obtained in this process. In order to convert the solid residue problem in this process, biochar was fabricated through a further chemical activation process. The carbon yield for BC-ZnCl_2_ and BC-H_3_PO_4_ is 35.9% and 32.4%, respectively. In addition, the chemicals could be recycled for activation in the next cycle (Figure 1). The as-prepared biochars were used for heavy metal ions removal. The adsorption amount for BC-ZnCl_2_ and BC-H_3_PO_4_ is 72.1 mg/g and 42.7 mg/g, respectively. 

Thus, BC-ZnCl_2_ is recognized as a better process for the concept. We believe the biochar preparation process expanded the conversion and utilization of biomass-derived solid residue.

### 4.3. Summary of Pb (II) Adsorption Capacities for Different Carbonaceous Materials

Table 4 summarizes the adsorption capacities for Pb (II) of different carbonaceous materials. Compared to other recently reported carbonaceous materials, the as-prepared BC-ZnCl_2_ and BC-H_3_PO_4_ samples exhibit excellent Pb (II) removal capacity via adsorption. As well as this, BC-ZnCl_2_ exhibited a low cost, effective biochar compared with other functionalized/modified biochars.

## 5. Conclusions

In this study, two biochar samples, BC-H_3_PO_4_ and BC-ZnCl_2_, were prepared from corn cob residue through a hydrothermal carbonization and subsequent chemical activation process. The biochars exhibit well-developed porous structures, with high BET surface areas (up to 790 m^2^/g for BC-ZnCl_2_) and acid groups. These are desirable features in regard to the removal of heavy metal ions from aqueous solutions.

The biochars show excellent adsorption capacities for heavy metal ions removal. The main mechanisms for metal ions removal include porous diffusion, electrostatic interactions and cation–π interactions. The BC-ZnCl_2_ sample demonstrates higher adsorption capacities than the BC-H_3_PO_4_ sample, due to its higher amount of acid groups (0.98 mmol/g vs 0.69 mmol/g), higher surface area (790 m^2^/g vs 680 m^2^/g), and higher porosity (0.74 cm^3^/g vs 0.65 cm^3^/g). 

The adsorption process is of a chemisorp nature, as indicated by the strong correlation between a pseudo-second-order model and the experimental data. Adsorption uptakes of Pb (II), Cd (II) and Cu (II) for the BC-ZnCl_2_ sample, which increase with the initial concentrations (0.5–5.0 mmol/L), are 23.1–72.1 mg/g, 6.8–55.6 mg/g and 8.2–30.5 mg/g, respectively. The isotherm adsorption data agree with the Langmuir model, these results support the conclusion that the developed biochars present a promising potential for heavy metal removal for water purification and treatment applications.

## Figures and Tables

**Table 1:** Summary of properties for Hydrochar, BC-H_3_PO_4_ and BC-ZnCl_2_.

Properties		Hydrochar	BC-H_3_PO_4_	BC-ZnCl_2_
pH_pzc_		9.8	3.5	4.6
BET surface Area (m^2^/g)		13.2	680	790
Pore volume (cm^3^/g)		0.068	0.65	0.74
Boehm Titration	Carbonyl/ (mmol/g)	-	0.095	0.21
	Carboxylic/ (mmol/g)	-	0.082	0.14
	Lactones/ (mmol/g)	-	0.065	0.090
	Phenolics/ (mmol/g)	-	0.45	0.54
	Basicity/ (mmol/g)	-	0.21	0.36

**Table 2:** Kinetic fitting parameters of pseudo-first-order (PFO), pseudo-second-order (PSO) and intra-particle diffusion (IP) models for BC-H_3_PO_4_ and BC-ZnCl_2_ removal of Pb (II).

C_o_	q_exp_	PFO			PSO			IPD		
		Q_1_	K_1_	R^2^	Q_2_	K_2_	R^2^	K_i_	C	R^2^
**BC-H_3_PO_4_**										
100 mg/L	11.5	9.7	0.045	0.854	11.8	0.0079	1.000	1.06	22.22	0.299
250 mg/L	25.8	28.2	0.064	0.914	24.9	0.0019	0.999	3.08	32.61	0.563
500 mg/L	39.7	38.0	0.061	0.898	39.9	0.0017	1.000	4.13	55.37	0.481
**BC-ZnCl_2_**										
150 mg/L	51.2	55.4	0.011	0.852	51.1	0.0092	1.000	3.45	41.74	0.408
370 mg/L	60.9	68.5	0.0093	0.842	60.7	0.0051	1.000	3.08	54.50	0.484
600 mg/L	65.3	69.6	0.0081	0.854	65.2	0.0057	0.999	4.13	89.56	0.377

**Table 3:** Isotherms fitting parameters of the Freundlich and Langmuir models for the BC-H_3_PO_4_ and BC-ZnCl_2_ removals of Pd (II), Cd (II) and Cu (II).

Biochar Type		Freundlich Model			Langmuir Model			
		K_F_ (L/mg)	n	R^2^	q_m_ (mg/g)	K (L/mg)	R^2^	R_L_
BC-H_3_PO_4_	Cu (II)	3.71	3.02	0.93	7.2	0.54	1.00	0.002
	Cd (II)	1.38	1.45	0.96	36.9	0.072	1.00	0.011
	Pb (II)	2.92	1.71	0.93	44.8	0.014	0.99	0.324
BC-ZnCl_2_	Cu (II)	6.37	3.15	0.73	27.5	0. 15	0.99	0.105
	Cd (II)	4.09	1.47	0.90	50.4	0.036	0.98	0.495
	Pb (II)	8.13	2.62	0.82	63.5	0.089	0.98	0.134

**Table 4:** Summary of the Pb (II) adsorption capacities of different carbonaceous materials.

Absorbent Used	Q Ads (mg/g)	References
Banana peel-activated carbon	34.5	[42]
Sugarcane cane biochar	86.9	[43]
Raw sugarcane bagasse biochar	81.9	[44]
Functionalized Porous lignin	188	[45]
Graphene/activated carbon composite	212	[28]
KMnO_4_-treated hickory wood biochar	153.1	[46]
This study (BC-H_3_PO_4_)	42.7	this study
This study (BC-ZnCl_2_)	72.1	this study

## Supplementary Materials

The following are available online at https://www.mdpi.com/1996-1944/13/5/1037/s1, Figure S1. SEM images of (a) 500×Hydrochar; (b) 5000×BC-H3PO4; (c) 4800×BC-ZnCl2. Figure S2. XRD of Hydrochar, BC-H_3_PO_4_ and BC-ZnCl_2_. Table S1. Kinetic fitting parameters of the PFO, PSO and IPD models for the BC-H_3_PO_4_ removal of Cd (II) and Cu (II). Table S2. Kinetic fitting parameters of the PFO, PSO and IPD models for the BC-ZnCl_2_ removal of Cd (II) and Cu (II).
