# Supplementary material for: Biochars from Lignin-rich Residue of Furfural Manufacturing Process for Heavy Metal Ions Remediation
Source: Materials (Basel). 2020 Feb 25;13(5):1037. doi: 10.3390/ma13051037 (PMC7084945; doi:10.3390/ma13051037)
Supplement: Supplementary file 1 [file materials-13-01037-s001.pdf]

materials

Supplementary

Biochars from Lignin-rich Residue of Furfural Manufacturing Process for Heavy Metal Ions Remediation

Baobin Wang [1,2,3], Miao Ran [2], Guigan Fang [1,2,*], Ting Wu [2] and Yonghao Ni [3,*]

[1] College of Light Industry Science and Engineering, Nanjing Forestry University, Nanjing 210037, China; wangbaobin0408@163.com
[2] Key Lab. of Biomass Energy and Material, Institute of Chemical Industry of Forestry Products, CAF, Nanjing 210042, China; ranmiaolhs@163.com (M.R.); wuting@icifp.cn (Y.N.)
[3] Limerick Pulp and Paper Centre and Department of Chemical Engineering, University of New Brunswick, Fredericton, New Brunswick, E3B 5A3, Canada
* Correspondence: fangguigan@icifp.cn (G.F.); yonghao@unb.ca (Y.N.); Tel: +1-506-451-6857 (Y.N.); Fax: +1-506-453-4767 (Y.N.)

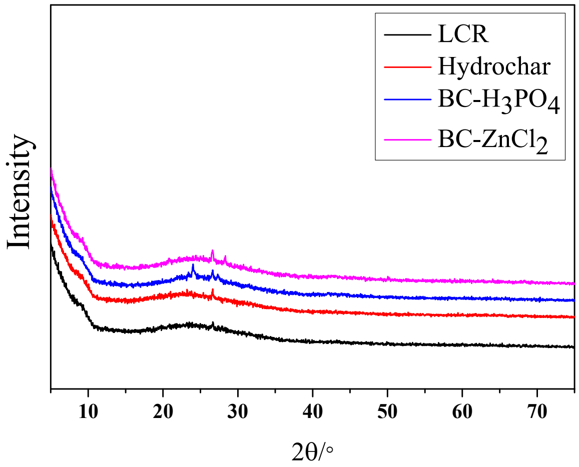

Figure S1. XRD of Hydrochar, BC-H_3PO_4 and BC-$ZnCl_2$.

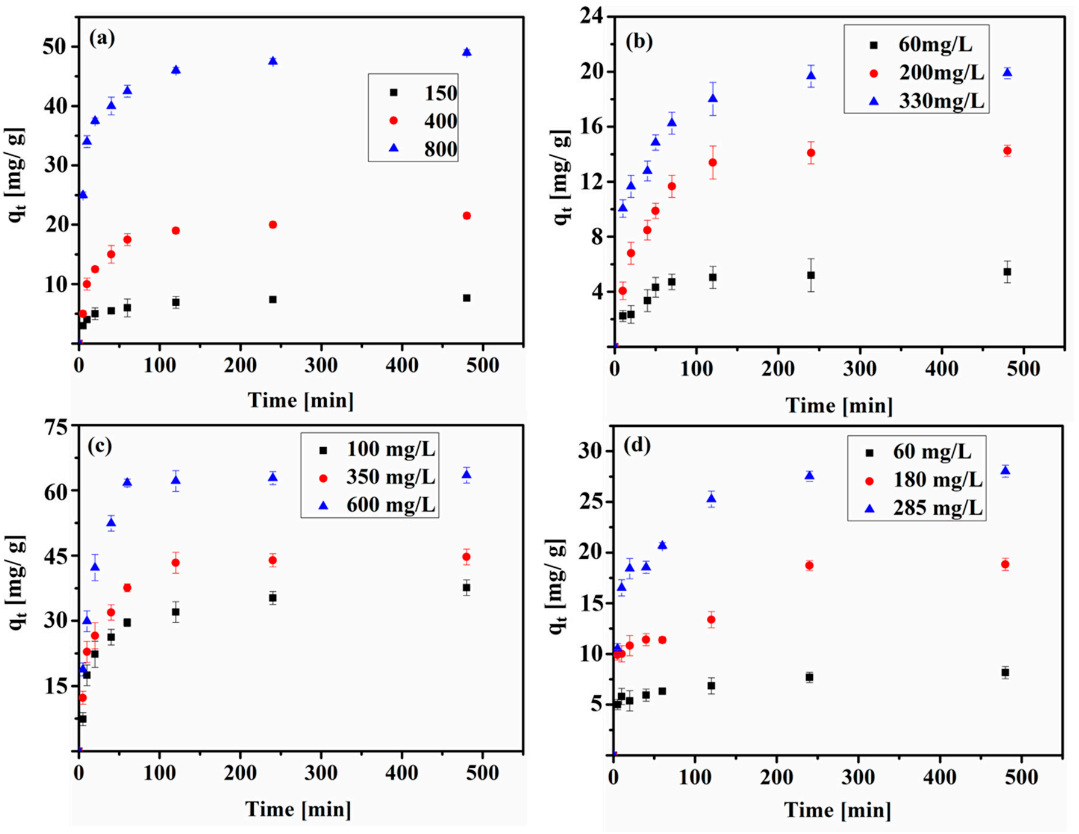

Figure S2. Kinetics analyses of the adsorption of Cd (II) on (**a**) BC-H_3PO_4, (**c**) BC-$ZnCl_2$ and the adsorption of Cu (II) on (**b**) BC-H_3PO_4, (**d**) BC-$ZnCl_2$ as a function of contact time.

Table S1. Kinetic fitting parameters of PFO, PSO and IPD model for BC-H_3PO_4 removal of Cd (II) and Cu (II).

C_o	q_{exp}	PFO			PSO			IPD		
		Q_1	K_1	R^2	Q_2	K_2	R^2	K_i	C	R^2
**Cd (II)**										
150 mg/L	5.3	7.5	0.032	0.645	5.0	0.0060	0.998	0.586	5.388	0.671
400 mg/L	20.1	24.3	0.50	0.768	19.6	0.0016	0.998	1.776	12.508	0.698
800 mg/L	48.2	37.9	0.047	0.636	47.8	0.0016	1.000	3.267	45.200	0.496
**Cu (II)**										
60 mg/L	4.8	3.7	0.03	0.550	4.8	0.136	0.999	0.266	2.401	0.635
200 mg/L	13.8	10.3	0.047	0.659	13.2	0.0185	0.999	0.745	5.492	0.695
330 mg/L	20.6	18.6	0.055	0.740	20.4	0.0733	0.999	0.903	9.856	0.624

materials

MDPI

Table 2. Kinetic fitting parameters of PFO, PSO and IPD model for BC-$ZnCl_2$ removal of Cd (II) and Cu (II).

C_o	q_{exp}	PFO			PSO			IPD		
		Q_1	K_1	R^2	Q_2	K_2	R^2	K_i	C	R^2
**Cd (II)**										
100 mg/L	33.1	36.9	0.086	0.940	32.9	0.005	0.998	5.22	5.001	0.707
350 mg/L	40.9	43.9	0.097	0.821	41.3	0.006	0.999	6.19	7.762	0.666
600 mg/L	62.1	69.2	0.113	0.745	62.7	0.006	0.999	8.61	6.567	0.570
**Cu (II)**										
60 mg/L	7.2	6.2	0.068	0.903	7.6	0.012	0.997	2.593	5.985	0.533
180 mg/L	18.4	15.4	0.103	0.879	18.9	0.032	0.985	6.919	6.527	0.730
285 mg/L	28.1	26.5	0.098	0.926	28.7	0.039	0.998	7.736	7.67	0.678

© 2020 by the authors. Submitted for possible open access publication under the terms and conditions of the Creative Commons Attribution (CC BY) license (http://creativecommons.org/licenses/by/4.0/).
